# Implementation strategies to increase seasonal influenza vaccination among adults: A rapid scoping review

**DOI:** 10.1080/21645515.2025.2481005

**Published:** 2025-04-07

**Authors:** Katherine Adams, Joanna Taliano, Ijeoma Okorie, Melissa Alvendia, Palak Patel, Shikha Garg, Larry W. Chang

**Affiliations:** a Influenza Division, Centers for Disease Control and Prevention, Atlanta, GA, USA; b Office of Science Quality and Library Services, Office of Science, Centers for Disease Control and Prevention, Atlanta, GA, USA; c Office of the Director, Centers for Disease Control and Prevention, Atlanta, GA, USA; d Department of Health Policy & Management, Johns Hopkins Bloomberg School of Public Health, Baltimore, Maryland; e Department of Epidemiology, Johns Hopkins Bloomberg School of Public Health, Baltimore, Maryland; f Department of Medicine, John Hopkins School of Medicine, Baltimore, Maryland; g Department of International Health, Johns Hopkins Bloomberg School of Public Health, Baltimore, Maryland

**Keywords:** Influenza, vaccines, implementation research, implementation science, scoping review

## Abstract

Many strategies have been applied to increase seasonal influenza vaccination; however, gaps in coverage remain. We synthesized the evidence on effectiveness of implementation strategies to increase seasonal influenza vaccination among U.S. adults. Studies performed from February 2010–August 2023 in the United States, focused on seasonal influenza vaccination, and measuring uptake and coverage were included. Guidance from Cochrane was followed. Interventions were mapped to Expert Recommendations for Implementing Change strategies. A total of 1,585 non-duplicate records were identified, full-text screening was performed for 353 records, and 51 studies met inclusion criteria. Among these studies, implementation strategies included those that engaged consumers, trained and educated stakeholders, and supported providers. Considerable heterogeneity was found in the study setting, populations, design, and methods. Substantial study variation limits the ability to conclude which strategies are most effective at increasing influenza vaccination uptake and coverage in U.S. adults.

## Introduction

### Significance

Seasonal influenza viruses contribute substantial annual morbidity and mortality in the United States.^1^ Vaccination is the primary public health intervention for the prevention of influenza illness, with the U.S. Centers for Disease Control and Prevention (CDC) Advisory Committee on Immunization Practices (ACIP) recommending yearly influenza vaccination for all adults.^2^ Vaccine protection against mild-to-moderate influenza illness can vary based on match between current season vaccine composition and circulating virus strains.^3,4^ However, estimates of population-level averted disease burden due to receipt of a seasonal influenza vaccine underscore consistently meaningful reductions in influenza-associated illness, medical visits, hospitalizations, and deaths.^5^

### Implementation gap

Despite the evidence on the benefits of vaccination, seasonal influenza vaccination coverage among U.S. adults remains lower (44.9% among adults ≥18 years during the 2023–2024 influenza season) than the U.S. Department of Health and Human Services’ Healthy People 2030 target of 70% national coverage.^6,7^ Moreover, coverage remains low based on certain clinical and demographic characteristics, contributing to increased risk of influenza complications among these populations.^8,9^ Among U.S. adults with preexisting high-risk medical conditions such as pulmonary and cardiovascular diseases, diabetes, and immunocompromising conditions, less than half have been found to be vaccinated against seasonal influenza.^10,11^ Previous analyses have also shown gaps in influenza vaccination coverage based on race and ethnicity, including among non-Hispanic Black, American Indian or Alaska Native, and Hispanic populations.^12–14^ Variation in coverage of influenza vaccines has been found based on pregnancy status, income, education, insurance, access to medical care, housing circumstances, living in rural areas, nativity, and language spoken.^15–21^ Additionally, lower influenza vaccination coverage has also been associated with social determinants of health, specifically increased social vulnerability including living in areas associated with lower socioeconomic status and certain housing conditions.^22–25^ These gaps suggest the influence of both individual determinants as well as social determinants of health in coverage of seasonal influenza vaccines.

### Evidence gaps

While influenza vaccine coverage gaps are well characterized, there has been no recent summary of the evidence on the effectiveness of implementation strategies to increase seasonal influenza vaccination, particularly among U.S. populations with historically low vaccine coverage. Implementation strategies are defined as approaches to increasing the adoption, implementation, and long-term sustainability of evidence-based interventions.^26^ These can include clinical and community-facing approaches such as media campaigns, community outreach, incentives to reduce treatment costs, and healthcare provider training. Implementation strategies can range from single to bundled/packaged, multi-component programs that target numerous barriers and facilitators of uptake of evidence-based interventions.^27^ However, a core component of implementation strategies is that they should be tailored to local, contextual factors to best address barriers and determinants of behavior change that impact successful implementation.^28^

Questions around implementation strategies for seasonal influenza vaccination include What implementation strategies have been used in the United States, and in which settings and populations? What assessments of the evidence supporting these interventions have been performed? What is the impact of implementation strategies on increasing influenza vaccination?

### Objectives

A rapid scoping review was performed to synthesize the evidence on effectiveness of implementation strategies aimed at increasing seasonal influenza vaccination among U.S. adults. Characterizing these approaches may enable researchers and implementing organizations to design strategies that increase seasonal influenza vaccination coverage.

## Methods

### Approach

A scoping review approach was selected in lieu of a formal systematic review in anticipation of the heterogeneity of evidence and approaches and to identify critical gaps in the research around effectiveness of implementation strategies.^29–33^Additionally, as the goal of the project was to characterize the evidence on effectiveness, a scoping review was determined as appropriate.

Guidance from Arksey and O’Malley (2007) on conducting scoping reviews was followed, along with recent (February 2024) Cochrane guidance for using rapid scoping review methods.^33–35^ A protocol for this rapid scoping review was developed, and all results were reported following the Preferred Reporting Items for Systematic Reviews and Meta-Analysis (PRISMA) checklist for scoping reviews (Supplementary Table 1).^36^

### Stage 1: identifying the research question

To clarify the review question, a PICO (population, intervention, comparison, outcomes) framework was used (Supplementary Table 2).^37^ Study outcome measures were designated as both vaccine uptake (i.e., number or proportion vaccinated during a prespecified time period) and coverage (i.e., overall proportion vaccinated) to encompass multiple measures of increases in vaccination. The following research question was developed: Among adults in the United States, what is the evidence on the effectiveness of implementation strategies to increase uptake and coverage of seasonal influenza vaccines?

### Stage 2: identifying relevant studies

In collaboration with a medical librarian (JT), a search was conducted on February 1, 2024 of multiple biomedical databases: MEDLINE, Embase, PsycInfo, Cochrane Library, Cumulative Index to Nursing and Allied Health Literature (CINAHL), and Scopus. A list of search terms (Supplementary Table 3) was developed by the authors to specifically capture studies addressing contextual determinants of low vaccine coverage, specifically those identified by the Centers for Disease Control and Prevention (CDC) and Agency for Toxic Substances and Disease Registry (ATSDR)’s Social Vulnerability Index.^38^ Additionally, the search used broad terms to capture implementation strategy concepts. The search was limited to articles published starting in 2010, and duplicates were excluded. References were reviewed for identification of any additional relevant articles. Search results were compiled in EndNote 20 (London, United Kingdom).

### Step 3: study selection – eligibility

The review was limited to full-text research studies published in peer-reviewed journals in the English language. Articles were excluded if they were published or conducted using data from prior to February 24, 2010 to reflect the universal recommendation of seasonal influenza vaccines for all adults by ACIP, and to focus on recently implemented strategies.^39^ If a study was not conducted in the United States, it was excluded. Articles not focused on seasonal influenza vaccination (e.g., influenza A(H1N1) pandemic, vaccination against non-influenza pathogens) were also excluded. Studies conducted exclusively on non-adult (<18 years) or non-human populations were excluded. Articles that were not original research studies (e.g., editorials, commentaries, opinion articles) were also excluded. Articles were excluded if they did not use outcomes measuring influenza vaccine uptake or coverage (e.g., other outcomes including vaccine effectiveness, safety, cost-effectiveness, increasing willingness/intention to be vaccinated, knowledge/perceptions). Finally, studies proposing but not reporting results of strategies that were implemented were excluded.

### Step 3: study selection – screening

Article screening was conducted using the Covidence (Melbourne, Australia) platform.^36^ A three-stage rapid screening process was followed (Supplementary Figure 1): 1) dual reviewer title and abstract screening of 40% of all records produced during the literature search; 2) single reviewer title and abstract screening of the remaining 60% of records; 3) single reviewer full-text screening.

Prior to screening, a tool was developed by the primary researcher (Supplementary Appendix A) following guidance on best practices for abstract screening.^40^ Pilot testing of the screening tool was performed on 30 randomly selected records. Calibration of screening and revision of the screening tool was then performed based on discussion between reviewers.

During the first screening stage, dual and independent title and abstract screening was performed. While Cochrane rapid scoping review guidance notes that 20% of records may be independently dual screened, due to the availability of an additional second screener, dual screening was increased to 40%.^35^ Two distinct sets, each consisting of 20% of non-duplicate records produced by the literature search, were randomly sampled using the “sample” command in Stata 18.0 (College Station, TX). These two sets of studies were imported into separate projects using the Covidence platform. Two reviewers (KA, MA) conducted dual, independent title and abstract screening of one set of studies, while two reviewers (KA, IO) screened the second set. Reviewer agreement was assessed, and percent agreement was considered acceptable (~85%) for both sets. Any disagreement among the two reviewers was resolved by a third reviewer (PP) prior to full text screening.

In the second screening stage, the primary researcher (KA) conducted title and abstract screening for the remaining 60% non-sampled records. Articles that were designated as appropriate for inclusion or those for which a clear determination could not be made moved to full text screening.

A third screening stage was then conducted where the primary researcher conducted full-text screening of articles determined to be potentially eligible during title and abstract screening. The nine eligibility criteria used during title and abstract screening remained unchanged; however, two additional eligibility criteria were added for full text screening: 1) whether a strategy to increase uptake or coverage of seasonal influenza vaccines was studied and 2) whether the strategy was implemented. These were added during the full text screening stage to reflect criteria anticipated to be ascertained primarily through full-text review. Finally, a second reviewer screened any articles excluded by the first reviewer during full-text screening to confirm ineligibility.

### Step 4: charting the data

Key variables were extracted by the primary researcher from all included studies using a structured data recording form (Supplementary Table 4) developed based on a template from the Joanna Briggs Institute Manual for Evidence Synthesis.^41^ Extracted variables included study authors, publication year, study geographic setting, study dates, study aims, study population, implementation setting, implementation strategy used, study design, methods, comparator group, outcome measures, and key findings.

### Step 5: collating, summarizing, and reporting the results

Extracted data were synthesized into results tables, and a narrative summary was developed. Study population characteristics – particularly those capturing dimensions of contextual determinants – were summarized if noted within the article text. Due to significant variation in reporting of details on study population demographics, these characteristics were exclusively narratively described.

Following data extraction, included studies were mapped to 73 unique implementation strategies developed by the Expert Recommendations for Implementing Change (ERIC), which used a modified Delphi process with a panel of implementation science experts to systematically harmonize nomenclature (Supplementary Table 5).^27^ Definitions for each implementation strategy as described by Powell et al. 2015 were used to map study descriptions of interventions to ERIC implementation strategies. For example, if the intervention was described as “delivery of a patient-centered educational pamphlet and benefit statement on influenza vaccination,” this was classified as “distribute educational materials” using the ERIC definition of “distribute educational materials (including guidelines, manuals, and toolkits) in person, by mail, and/or electronically.” Notably, many studies included multi-component interventions, which were classified into multiple ERIC strategies.

Following classification, identified implementation strategies were then categorized into clusters described by Waltz et al. 2015 reflecting nine core concepts. These concepts include *using evaluative and iterative strategies, providing interactive assistance, adapting and tailoring to the context, developing stakeholder interrelationships, training and educating stakeholders, supporting clinicians* (reframed as “*supporting providers*” to encompass both medical staff and pharmacists),* engaging consumers, utilizing financial strategies*, and *changing infrastructure*.^42^ These clusters were used to narratively summarize study findings.

Study results were summarized to broadly characterize the state of the evidence on effectiveness of implementation strategies to increase vaccination. Four discrete results categories were defined: 1) *positive* study findings, i.e., those reporting a statistically significant (as designated by individual studies) effect of the implementation strategy on increasing vaccination; 2) *non-significant*, i.e., no statistically significant effect found; 3) *negative*, i.e., statistically significant effect of the strategy on decreasing vaccination; and 4) *mixed*, defined as studies with overall results that were non-significant or negative but positive results among certain populations or study cohorts. If no significance test was performed by a study but an increase in vaccination was reported, the study was considered to have positive results. The total number of studies (ranging from 0 to 14) included from each implementation strategy category were visualized in a heat map.

## Results

### Search results

**Figure 1.**
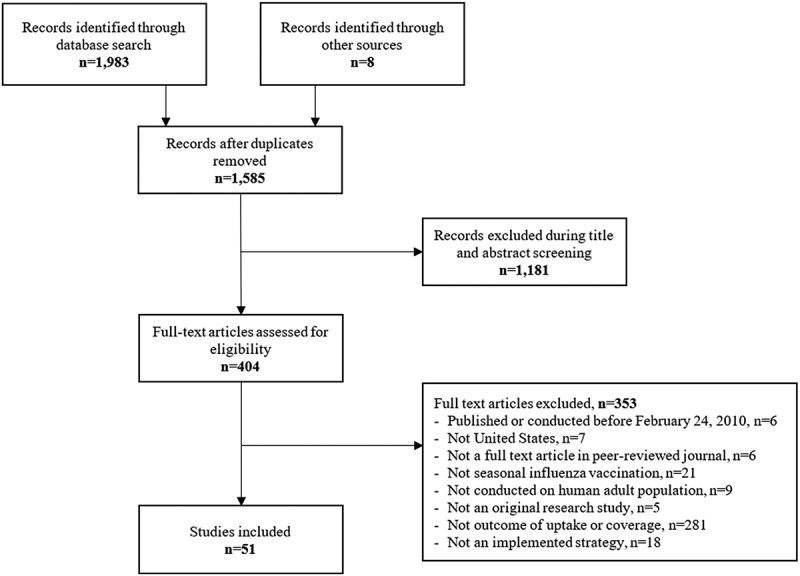
PRISMA diagram.

The review selection and exclusion process is described in Figure 1. 1,585 records were eligible for title and abstract screening, of which 404 (25%) were eligible for full-text screening. The primary reason for exclusion during full-text screening was that the study did not focus on uptake or coverage of vaccines. A total of 51 studies were included in data extraction.

### Study characteristics

**Figure 2.**
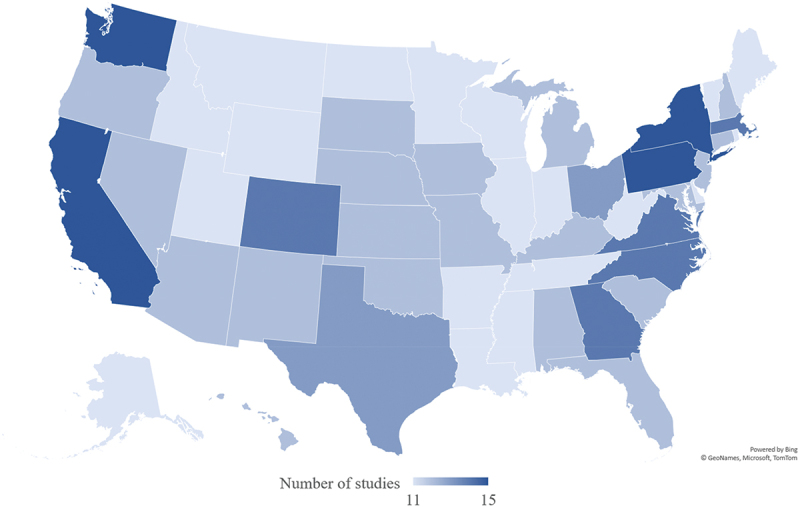
Geographic distribution of included studies across U.S. states.

**Figure 3.**
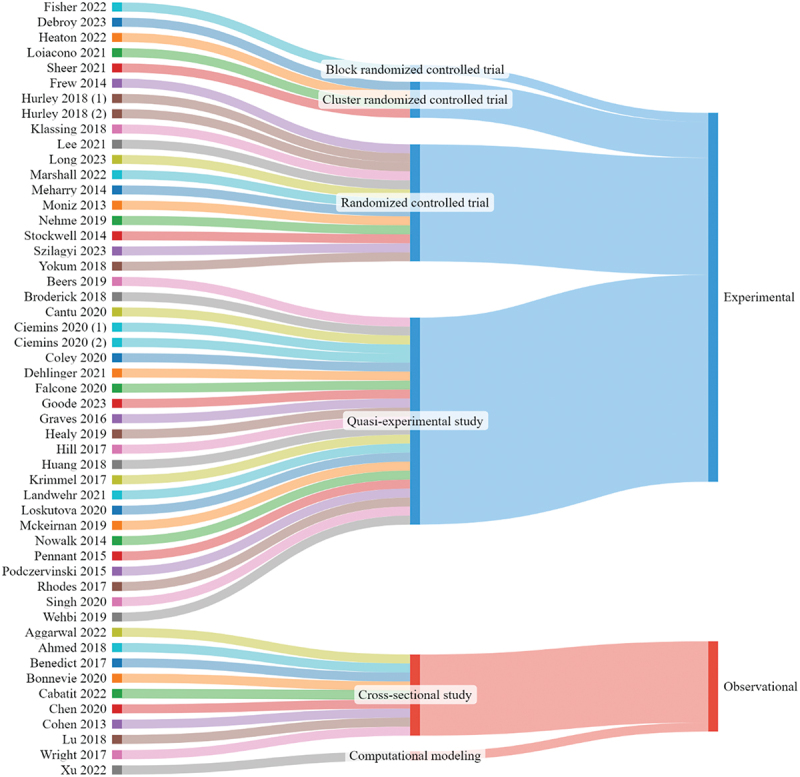
Included studies and study design.

Studies included in the analysis were published between 2013 and 2023 (Table 1, Table 2). Geographic setting varied, with 11 studies conducted across the United States^43,44,46,51,72,73,76,77,87,92,93^ and five across multiple U.S. states (Figure 2).^47,52,53,74,90^ Most implementation strategies (N = 30) were conducted within clinical locations (health care organizations and ambulatory care settings, such as outpatient clinics, emergency departments, free clinics, specialty services).^45,46,48,50,52–54,56–59,61,63–65,67,68,70,72,74–77,79,80,82,83,88,89,91^ Most of the studies (N = 41) used experimental designs (Figure 3), with 23 quasi-experimental approaches^45,48,50,52,53,55,57,58,61–63,65,66,70,71,75,78,82–85,87,90^ and 18 randomized controlled trials or using randomization procedures (i.e., four cluster randomized trials and one block randomized trial).^56,59,60,64,67–69,72–74,77,79–81,86,88,89,93^ Among all included studies, 10 used observational designs, with nine cross-sectional studies,^43,44,46,47,49,51,54,76,91^ and one using computational modeling.^92^ Most studies (N = 28) used single-component strategies,^43,44,46,47,49,51,54,59,60,64,71–74,76–80,85–93^ while 23 were multi-component.^45,48,50,52,53,55–58,61–63,65–70,75,81–84^

For studies conducted nationwide, each state was counted once per study. Specific geographic locations were unable to be determined for 2 studies (Ciemins et al.,^52^ Ciemins et al.^53^).

**Table 1.** Study characteristics.

Characteristic	Total N = 51 studies
Publication year	
2013	2 (4%)
2014	4 (8%)
2015	2 (4%)
2016	1 (2%)
2017	5 (10%)
2018	8 (16%)
2019	5 (10%)
2020	9 (18%)
2021	5 (10%)
2022	6 (12%)
2023	4 (8%)
2024	0 (0%)
Setting^a^	
United States, nationwide	11 (22%)
Multi-state	5 (10%)
Midwest	6 (12%)
Northeast	12 (24%)
Pacific West	5 (10%)
Rocky Mountain	2 (4%)
Southeast	8 (16%)
Southwest	2 (4%)
Implementation setting^a^	
Clinical	30 (59%)
College campus	1 (2%)
Community	1 (2%)
Online	2 (4%)
Pharmacy	12 (24%)
Workplace	3 (6%)
Other/unclear	6 (12%)
Study design/methods	
Computational modeling	1 (2%)
Cross-sectional	9 (18%)
Quasi-experimental	23 (45%)
Randomized trials	18 (35%)
Implementation category^b^	
Develop stakeholder interrelationships	4 (8%)
Engage consumers	22 (43%)
Support providers	18 (35%)
Train and educate stakeholders	20 (39%)
Use evaluative and iterative strategies	4 (8%)
Utilize financial strategies	6 (12%)
Implementation approach^c^	
Single-component strategy	28 (55%)
Multi-component strategy	23 (45%)

^a^U.S. geographical regions included: Midwest = Illinois, Indiana, Iowa, Kansas, Michigan, Minnesota, Missouri, Nebraska, North Dakota, Ohio, South Dakota, Wisconsin; Northeast = Connecticut, Delaware, Maine, Maryland, Massachusetts, New Jersey, New Hampshire, New York, Pennsylvania, Rhode Island, Vermont; Pacific West = Alaska, California, Hawaii, Oregon, Washington; Rocky Mountain = Colorado, Idaho, Montana, Nevada, Utah, Wyoming; Southeast = Alabama, Arkansas, Florida, Georgia, Kentucky, Louisiana, Mississippi, North Carolina, South Carolina, Tennessee, Virginia, West Virginia; Southwest = Arizona, New Mexico, Oklahoma, Texas

^b^Not mutually exclusive

^c^Single-component strategies were defined as those delivering a single intervention to a study population; multi-component strategies were defined as those with two or more interventions delivered to the same study population.

**Table 2.** Study details.

Authors, year	Setting	Study dates	Study design	Population	Implementation setting	Strategy description	CFIR strategy	Outcome	Comparator	Results
Aggarwal et al.^43^	United States nationwide	December 5, 2021–April 10, 2022	Cross-sectional study	2622 older (≥65 years) adults with low income and enrolled in Medicare Advantage	National	Expanded Medicare benefits (Medicare Advantage)	Use capitated payments	Odds of vaccination	Traditional Medicare	No significant differences between groups in likelihood of receipt of an influenza vaccination in the past year (66.3% versus 63.8%; aOR, 1.16 [95% CI, 0.93–1.45]).
Ahmed et al.^44^	United States nationwide	March–April 2015	Cross-sectional study	838 White and 819 African Americans adult participants	Online	Use of social media for health information	Use mass media	Odds of vaccination	Participants who did not use social media for health information	Users of Twitter (OR 4.41, 95% CI: 1.43–13.60) and Facebook (OR 1.66, 95% CI: 1.01–2.72) for health information were more likely to be vaccinated compared to those who do not use these platforms for health information.
Beers et al.^45^	Regional rural area in Midwest	2018–2019 influenza season	Quasi-experimental study	54 homeless adults either residing in a homeless shelter or attending a meal site; 45 employees at a primary care clinic	Clinic, community	Provider education, patient info sheet developed with stakeholder input	Conduct educational meetings; Distribute educational materials	Vaccination rate	Prior season (2017–2018)	Influenza vaccination rates improved at the meal site but decreased from 24.77% in 2017–2018 to 23.85% in 2018–2019 at the homeless clinic.
Benedict et al.^46^	United States nationwide	March 1–29, 2012	Cross-sectional study	15,630 respondents to National Flu Survey (NFS)	Clinic	Provider recommendation & offer of vaccination	Involve patients/consumers and family members	Prevalence of vaccination	No recommendation or offer	Those reporting both receiving a provider recommendation and vaccination offer were 1.76 times more likely to be vaccinated; those reporting receiving a provider recommendation but no vaccination offer were 1.72 times more likely to be vaccinated.
Bonnevie et al.^47^	Campaign areas: Northern California, Southern California, Colorado, Georgia, Hawaii, Maryland, Virginia, and Washington D.C., Oregon, and Washington. Control areas: Alabama, Arkansas, Nevada, New Mexico, New York, Oklahoma, Pennsylvania, and South Carolina	October 2018–March 2019	Cross-sectional study	117 recruited influencers	Online	Exposure to social media micro influencer campaigns	Use mass media; Intervene with patients/consumers to enhance uptake & adherence; Identify and prepare champions	Vaccination rate	Control group	45% in the campaign group received the vaccine versus 42% in the control group, with results non-significant.
Broderick et al.^48^	New York City	September 1, 2014–August 2015	Quasi-experimental study	228 outpatients with rheumatoid arthritis	Clinic	Provider multimodal intervention: education session, electronic medical record alerts, and weekly provider-specific e-mail reminders	Conduct educational meetings; Distribute educational materials; Remind clinicians; Facilitate relay of clinical data to providers	Missed opportunities for influenza vaccination (refusals not included)	Pre-intervention baseline	Pre-intervention missed opportunities were 47%, compared to post-intervention reduction to 23% (p < .001). However, there were no improvements among certain populations such as non-Hispanic Black patients and non-English speakers.
Cabatit et al.^49^	Southwest Virginia	2020–2021 influenza season	Cross-sectional study	Adult (≥18 years) patients at 2 large, national chain community pharmacies; 111 patient survey participants	Pharmacy	Text messages from pharmacy on influenza vaccine availability, information to schedule appointment	Intervene with patients/consumers to enhance uptake & adherence	Percentage vaccinated	Prior season (2019–2020)	17.45% increase in influenza vaccines administered among patients who received text messages; 13.22% overall increase in influenza vaccines administered during the 2020–2021 season compared with the 2019–2020 season.
Cantu et al.^50^	San Antonio, Texas	July–September 2018	Quasi-experimental study	1690 adult patients at a suburban academic primary care practice	Clinic	Patient vaccine questionnaire for waiting room + bundled intervention (revised clinical workflow, increased staff access to state vaccination registry and EMR, and patient education)	Facilitate relay of clinical data to providers; Develop and implement tools for quality monitoring; Distribute educational materials	Vaccination rate	Pre-intervention baseline	Questionnaire did not result in improved immunization rates, but intervention bundle showed sustained improvements.
Chen et al.^51^	United States nationwide	2010–2017	Cross-sectional study	Older adults (≥65 years)	National	Mass media influenza-related news reports	Use mass media	Vaccination rate	Pre-intervention baseline	100 additional influenza news media reports published during October increased vaccination uptake rate among older adults by 0.3 percentage points. This association was also found for January, but not November or December.
Ciemins et al.^52^	7 U.S. states	2016–2018	Quasi-experimental study	Approximately 858,000 adult patients receiving health care services at 9 large US health care organizations	Clinic, pharmacy	Learning collaborative model (standing orders, use of state vaccination and EHR registries, patient and provider education, patient outreach, alerts on health maintenance and best practices, working with specialists and pharmacies, selecting provider champions, and examining high performers)	Facilitate relay of clinical data to providers; Create a learning collaborative; Distribute educational materials; Intervene with patients/consumers to enhance uptake & adherence; Identify and prepare champions; Build a coalition	Vaccination rate	Nonparticipating provider population	9.5% (p < .001) improvement in influenza vaccination rates in individual health systems; 2.4% (p < .001) increase in rates for one intervention cohort.
Ciemins et al.^53^	7 U.S. states	2013–2016	Quasi-experimental study	Approximately 595,000 adult patients receiving health care services at 7 large US health care organizations	Clinic	Learning collaborative model (convening of expert advisory committees, monthly webinars, best practice sharing and coaching during site visits, provider education, goal setting, case studies, and clinical outcome measurement.	Create a learning collaborative; Distribute educational materials; Visit other sites; Use advisory boards and workgroups	Vaccination rate	Nonintervention sites	12.1% (p < .01) improvement in influenza vaccination rates in individual health systems; non-significant effects for overall average treatment effect of intervention.
Cohen et al.^54^	Brooklyn, New York	August 2010 to May 2011	Cross-sectional study	149 “treat-and-release” patients (English-speaking, ≥18 years old) from one emergency department	Clinic	Pharmacy-based influenza vaccination program (screening, standing orders and clinical workflow design)	Revise professional roles	Percentage vaccinated	Prior 8 years	41% of patients received the vaccine in the ED, a 4-fold increase from the previous 8 years.
Coley et al.^55^	Western Pennsylvania	September 1, 2016–August 31, 2017	Quasi-experimental study	99 regional supermarket chain pharmacies	Pharmacy	Automated notifications to pharmacists on patient eligibility and motivational interviewing processes	Remind clinicians	Percentage vaccinated	Prior year (2015–2016)	45% increase in influenza vaccinations compared with previous year.
Debroy et al.^56^	Atlanta, Georgia	October 2018 and April 2019	Cluster randomized controlled trial	88 primary care teams at the Atlanta Veterans Affairs Medical Center who saw 28,941 patients during the study period	Clinic	Modified clinical reminder (bundled electronic medical record reminders, patient vaccination history dashboard, provider talking point prompts)	Facilitate relay of clinical data to providers; Remind clinicians	Vaccination rate	Control group	Increased vaccination in intervention group but non-significant: +1.6 (95% CI: 1.3–4.4, p = .28); no reduction in vaccination rate disparities based on race.
Dehlinger et al.^57^	Cincinnati, Ohio tri-state region	2019–2020 influenza season	Quasi-experimental study	2967 pregnant women at an urban, Midwestern, academic health care facility	Clinic	Multi-strategy bundle: patient education (delivered during consent form administration), staff education, staff reminders, EHR prompts	Distribute educational materials; Facilitate relay of clinical data to providers; Remind clinicians	Vaccination rate	Prior season (2018–2019)	Higher rate of influenza vaccination during 2019–2020 season (63%) compared with 2018–2019 (59%) season (p = .01).
Falcone et al.^58^	Southeast Florida	September 2018–January 2019	Quasi-experimental study	Patients and providers at a free clinic in southeast Florida	Clinic	Bundled interventions (education to employees, mass communication to patients, improved workflow, and improved access)	Conduct educational meetings; Distribute educational materials; Intervene with patients/consumers to enhance uptake & adherence; Alter patient/consumer fees	Vaccination rate	Prior season (2017–2018)	597% increase in vaccination rate from 2017–2018 (n = 35) to 2018–2019 (n = 244).
Fisher et al.^59^	Central Massachusetts	January 13, 2021–March 31, 2021	Block randomized controlled trial	36,920 households of patients from Reliant Medical Group clinics	Clinic	Household-based patient outreach pilot program using tailored EHR communication or interactive voice response telephone calls	Intervene with patients/consumers to enhance uptake & adherence	Percentage vaccinated	Pre-intervention baseline and control group	3.3% increase in vaccination compared to pre-program start, but no significant differences in between study arms.
Frew et al.^60^	Atlanta, Georgia	September 2011–May 2013	RCT	276 pregnant minority women	Other	One of three vaccine messages (standard information sheet, “gain-frame” messaging, “loss-frame” messaging)	Develop educational materials	Odds of vaccination	Control group	Neither intervention messaging (gain- or loss-framed) had significant association with increased likelihood of influenza immunization during pregnancy
Goode et al.^61^	Richmond, Virginia	2020–2021 influenza season	Quasi-experimental study	1,269 adult patients at a health care provider for underserved populations (homeless, uninsured)	Clinic, pharmacy	Multicomponent quality improvement initiative (staff education and training, patient education and messaging, clinical process and workflow redesign, EHR alerts to provider) led by a pharmacist champion	Conduct educational meetings; Distribute educational materials; Remind clinicians; Revise professional roles	Percentage vaccinated	Prior season (2019–2020)	42% increase in influenza vaccinations from prior influenza vaccination season.
Graves et al.^62^	Seattle, Washington	Fall 2012	Quasi-experimental study	Employees at eleven restaurants	Workplace	Educational materials to beneficiaries, on-site vaccination events, free vaccinations	Alter patient/consumer fees; Distribute educational materials	Vaccination rate	Pre-intervention baseline	Proportion vaccinated increased from 26% to 46% (aOR 2.33, 95% CI: 1.69–3.22)
Healy et al.^63^	Sioux Falls, South Dakota	2016–2017 influenza season	Quasi-experimental study	Patients at two outpatient family medicine clinics	Clinic	Quality initiative (improved clinical workflow, physician and staff education, patient posters and handouts)	Conduct educational meetings; Distribute educational materials; Revise professional roles	Vaccination rate	Prior two seasons (2014–2015 and 2015–2016)	Influenza vaccination rates increased from 35% to 53% (p < .01)
Heaton et al.^64^	California	April 1, 2019–March 31, 2020	Cluster randomized controlled trial	36 districts in California comprising 501 national chain pharmacies	Clinic, pharmacy	Pharmacist access to immunization information systems	Facilitate relay of clinical data to providers	Vaccination rate	Pre-intervention baseline	No significant differences in influenza vaccination rates among patients 19–64 years (adjusted RRR 0.99 [95% CI 0.83–1.17]) and ≥65 years (1.02 [0.86–1.22]).
Hill et al.^65^	Kansas City, Kansas	October 2011	Quasi-experimental study	142 patients during weekend days at the 32-bed Cardiovascular Progressive Care (CVP) unit at the University of Kansas Health-System	Clinic	Pharmacy technician intervention (review of immunization status, generation of eligibility list, relay of data to nursing staff), standing order program for nurses	Revise professional roles	Vaccination rate	Pre-intervention baseline, control group	Satistically significant (p = .001) increase in vaccination rate from 72.2% (52/72) in the control group to 92.9% (65/70) in the intervention group
Huang et al.^66^	Cambridge, Massachusetts	2013–2014 to 2015–2016 influenza seasons	Quasi-experimental study	6,650 northeastern U.S. university undergraduate students	College campus	Initial intervention: community health worker peer outreach; enhanced intervention: addition of personalized social media campaign	Identify and prepare champions; Use mass media	Vaccination rate	Prior seasons (2011–2012, 2012–2013), control group	Vaccination increased 66% (IRR = 1.66, 95% CI: 1.39–1.97) during the initial intervention, and 85% (IRR = 1.85, 95% CI: 1.75–1.96) during the enhanced intervention.
Hurley et al.^67^	Denver, Colorado	October 2015–April 2016	RCT	47,268 patients at urban safety-net healthcare system	Clinic	Centralized reminder/recall using an Immunization Information System; posters promoting adult vaccinations for patient care areas	Facilitate relay of clinical data to providers; remind clinicians; Distribute educational materials	Vaccination rate	Control group	Among patients ≥65 years, 32.0% in intervention group received a vaccine versus 28.6% in control group (p ≤ .01). Higher proportion also receiving flu vaccine in the younger age (19–64 years) strata.
Hurley et al.^68^	Denver metropolitan area and Northeastern Colorado	September 2016–April 2017 (RCT study period)	RCT	15,807 adult Medicaid patients	Clinic	Vaccine reminder/recalls; posters promoting adult vaccinations for patient care areas	Intervene with patients/consumers to enhance uptake & adherence; Distribute educational materials	Vaccination rate	Control group	No significant differences in receipt of influenza vaccine between study arms (19–64 year-olds: 20.1% intervention versus 19.0% control (p = .08); ≥65 year-olds: 38.0% intervention versus 39.5% control (p = .72)
Klassing et al.^69^	Kansas City, Missouri metro area	October–November 2014	RCT	831 adult patients with asthma and/or chronic obstructive pulmonary disease who filled prescriptions at one of three community pharmacies	Pharmacy	A personal phone call or standardized mailed letter recommending influenza and pneumococcal vaccinations	Intervene with patients/consumers to enhance uptake & adherence	Vaccination rate	Control group	The influenza vaccine was administered to 56 (72.7%) patients in the phone call group, 55 (87.3%) patients in the letter group, and 62 (88.6%) patients in the control group (p = .019).
Krimmel et al.^70^	New Brunswick, New Jersey	2015–2016 influenza season	Quasi-experimental study	48 transplantation recipients at Rutger’s Cancer Institute	Clinic	Tool kit with an education pamphlet and financial incentive (voucher), and a reminder letter	Alter patient/consumer fees; Distribute educational materials	Vaccination rate	Prior season (2014–2015)	Improvement in vaccination adherence from 63% (2014–2015 season) to 89% (2015–2016 season).
Landwehr et al.^71^	Cincinnati, Ohio	2019–2020 flu season	Quasi-experimental study	170 full-time, insured employees at a large financial group	Workplace	Educational handouts at an onsite health fair or lunch and learn	Distribute educational materials	Number of vaccinations	Prior season (2018–2019)	Increase from 337 to 406 vaccines administered during the same period
Lee et al.^72^	United States nationwide	September 2018–April 2019	RCT	31,404 U.S. adults with diabetes	Clinic	Monthly messages through an online health platform	Intervene with patients/consumers to enhance uptake & adherence	Vaccination rate	Control group	64.2% vaccinated in intervention versus 61.1% in the control arm (p = .0013); 8% increase in vaccination (p < .0001) if intervention messages completed.
Loiacono et al.^73^	United States nationwide	September 1, 2019–February 29, 2020	Cluster randomized controlled trial	4589 pharmacies	Pharmacy	Behavioral peer comparison intervention (electronic communications to pharmacies on performance compared to peers)	Audit and provide feedback; Develop and implement tools for quality monitoring	Percentage of additional doses	Control group	Overall non-significant findings: 4.1% (95% CI 0.1%–8.3%) additional doses administered in intervention group versus controls. But historically low-performing, large-format pharmacies had significant results (6.1% [95% CI 0.5%–11.9%] additional doses).
Long et al.^74^	Los Angeles, California and Chapel Hill, North Carolina	September 2019–March 2020	RCT	1056 patients with inflammatory bowel disease	Clinic	Preventive health videos versus text messages developed using a patient-centered approach	Distribute educational materials; Intervene with patients/consumers to enhance uptake & adherence	Percentage vaccinated	Prior season (2018–2019)	While there was an increase in intention to receive the vaccine compared to the previous year for both groups, there was no difference in actual receipt of vaccines for both groups (text message 55% to 57% [p = .23], video message 59% to 63% [p = .07]) compared to prior season.
Loskutova et al.^75^	Wilmington, North Carolina	July 2015–August 2016	Quasi-experimental study	43 general internal medicine and family medicine providers at ten sites within one health care organization	Clinic	Provider reminders, quarterly provider-level performance reports, provider education, patient visual aid materials, and standing orders	Remind clinicians; Audit and provide feedback; Distribute educational materials; Revise professional roles	Vaccination rate	Prior season (2014–2015)	Influenza vaccination rate increased 6.9 percentage points (p = .001), from 44.4 ± 16.7 to 51.3% ± 12.9%.
Lu et al.^76^	United States nationwide	2016–17 influenza season	Cross-sectional study	4305 survey participants	Clinic	Provider recommendation and offer of vaccination	Involve patients/consumers and family members	Odds of vaccination	Study groups	Higher vaccination coverage for those reporting provider recommendation and offer (66.6%) than those who reported that a provider only recommended but did not offer (48.4%), those who did not receive a recommendation or offer (32.0%), and those who did not visit a doctor (28.8%).
Marshall et al.^77^	United States nationwide	2020–2021 influenza season	RCT	49,138 adults with cardiovascular disease who were members of a mobile health platform	Clinic	Digital intervention messages to patients promoting vaccination	Intervene with patients/consumers to enhance uptake & adherence	Vaccination rate	Control group	Significantly higher vaccination rate in the intervention group (61.31%) than the control group (59.25%) (p = .03).
Mckeirnan et al.^78^	Washington state	July 2018–June 2019	Quasi-experimental study	7 pharmacy technicians at an Indian Health Services federal facility	Pharmacy	Pharmacist immunization training	Conduct educational meetings	Number of vaccinations	Previous 10 fiscal years (2009–2018)	During fiscal year 2019, 885 more flu shots were given than any of the previous 10 fiscal years.
Meharry et al.^79^	Connecticut	2011–2012 season	RCT	135 pregnant women at three locations	Clinic	Patient-centered pamphlet and benefit statement	Distribute educational materials	Vaccination rate	Control group	Higher vaccination in the pamphlet group (72.9%) and the pamphlet/benefit statement group (86.1%) than the control group (46.9%) (both p < .05).
Moniz et al.^80^	Pittsburgh, Pennsylvania	2010–2011 and 2011–2012 influenza seasons	RCT	216 obstetric patients at less than 28 weeks of gestation receiving prenatal care	Clinic	Weekly educational text messages	Intervene with patients/consumers to enhance uptake & adherence	Vaccination rate	Control group	No significant difference in vaccination between general message group (31%) compared with flu message group (33%) (difference 1.7%, 95% CI: −11.1–14.5%).
Nehme et al.^81^	Central Texas	September–November 2017	RCT	25,649 members of an Affordable Care Act insurance plan	Other	E-Mail and text messages, mailed letters	Intervene with patients/consumers to enhance uptake & adherence	Vaccination rate	Control group	Higher vaccination in the text message and mail group versus the no message group (rate difference: 2.5%; 95% CI: 1.4–3.6) and text message only group (rate difference: 1.6%; 95% CI: 0.5–2.8).
Nowalk et al.^82^	Allegheny County, Pennsylvania	June 2011–May 2012	Quasi-experimental study	5,592 patients at four urban primary care practices	Clinic	Toolkit to support standing order program	Develop educational materials; Distribute educational materials; Revise professional roles	Vaccination rate	Prior season (2010–2011)	Three of four sites had increases in influenza vaccination rates, and overall vaccination increased (22% in 2010–2011 versus 33% in 2011–2012; p < .001). However, vaccination only increased among high-risk adults 18–64 years (25% versus 40%, p = .02) and not among older adults ≥65 years (44% versus 52%, p = .26)
Pennant et al.^83^	Boston, Massachusetts	February 2009–January 2015	Quasi-experimental study	For influenza vaccination, patients of two specialties (allergy and infectious disease) with high-risk populations at Brigham and Women’s Hospital ambulatory specialty practices	Clinic	3 strategies for quality improvement: physician reminders, patient letters, and a nurse-driven model	Remind clinicians; Revise professional roles; Intervene with patients/consumers to enhance uptake & adherence	Vaccination rate	2011 versus 2014	Increases in vaccination rates across both specialties: allergy 59% in 2011 and 64% in 2014; infectious disease 74% in 2011 to 86% in 2014.
Podczervinski et al.^84^	Seattle, Washington	2010–2012	Quasi-experimental study	Approximately 1500 employees and clinical staff at a large comprehensive cancer care center	Workplace	Incentive-based strategy and penalty-based strategy (employee education, disciplinary action)	Alter incentive/allowance structures; Distribute educational materials; Develop disincentives	Vaccination rate	Pre-intervention baseline (2010)	Penalty-based strategy improved vaccination rates more than incentive-based strategy: 2010 versus 2011 (p = .0001); 2010 versus 2012 (p < .0001).
Rhodes et al.^85^	Rural North Carolina	January–February 2016	Quasi-experimental study	631 screenings at 5 independent community pharmacy locations	Pharmacy	A pharmacist vaccine screening tool and documentation form	Develop and implement tools for quality monitoring	Number of vaccinations	Same period during previous year (January–February 2015)	11 influenza vaccines were administered compared to 0 during the same time period in the previous year.
Sheer et al.^86^	Lexington, Frankfort and Louisville, Kentucky	January–June 2017	Cluster randomized controlled trial	2798 patients in influenza cohort	Pharmacy	Pharmacist reports on patient gaps in influenza vaccination	Facilitate relay of clinical data to providers	Percentage vaccinated	Control group	Higher odds of delivering influenza vaccines in intervention pharmacies than control (OR, 2.18; 95% CI, 1.37–3.46).
Singh et al.^87^	United States nationwide	2015–2016 and 2016–2017 flu seasons	Quasi-experimental study	Nationwide pharmacy chain	Pharmacy	Voucher for free flu vaccine	Alter patient/consumer fees	Voucher redemption, estimated averted influenza cases	Between study years	600,000 vouchers with a redemption rate of 52.3%, resulting in 314,033 flu vaccinations in 2015–2016; during 2016–2017 the redemption rate increased to 87.2%. A higher estimated averted number of influenza cases was found during 2016–2017 than 2015–2016.
Stockwell et al.^88^	New York City	September–December 2011	RCT	1187 obstetric patients at 5 community-based clinics serving low-income populations	Clinic	Vaccine text message reminders	Intervene with patients/consumers to enhance uptake & adherence	Odds of vaccination	Control group	Those in the intervention group were 30% more likely to be vaccinated
Szilagyi et al.^89^	Los Angeles, California	2020–2021 influenza season	RCT	213,773 patients across multiple specialties (primary care internal medicine, pediatrics, family medicine)	Clinic	Electronic health record patient portal messages, reminders, and scheduling	Intervene with patients/consumers to enhance uptake & adherence	Vaccination rate	Control group	No difference between study arms in vaccination rate (p > .017).
Wehbi et al.^90^	Nebraska and Iowa	October 2016–September 2017	Quasi-experimental study	159 community pharmacies	Pharmacy	Bidirectional pharmacy-based technology platform	Facilitate relay of clinical data to providers	Vaccination rate	Prior season (2015–2016)	37% increase in influenza vaccination rate
Wright et al.^91^	Amherst and Concord, New Hampshire	2014–2015 influenza season	Cross-sectional study	212 adults patients (aged ≥65 years) at two nurse practitioner-managed clinics	Clinic	Pre-visit preventative care planning (other strategies used for herpes zoster and Tdap)	Revise professional roles; Facilitate relay of clinical data to providers	Vaccination rate	National vaccination rates	Higher vaccination rates in nurse practitioner-managed clinics compared with national rates (77.8% versus 71.5%)
Xu et al.^92^	United States nationwide	January 1, 2011–December 31, 2018	Computational modeling	3951 articles published in 44 newspapers	Other	Daily and weekly U.S. newspaper articles with “flu” or “influenza” in the headline	Use mass media	Vaccination rate	High- versus low-exposure states	Newspaper articles on flu prevention tips, influenza caused deaths or illnesses stories, things to know about flu vaccine, and public accountability showed effectiveness at increasing adult influenza vaccine uptake.
Yokum et al.^93^	United States nationwide	September 2014–May 2015	RCT	228,000 Medicare recipients who were ≥66 years	Other	Motivational mailed letters from National Vaccine Program Office and the acting U.S. Surgeon General	Intervene with patients/consumers to enhance uptake & adherence	Vaccination rate	Control group	Percent vaccinated increased in all study arms receiving motivational letters (p < .001), but no significant differences were observed based on type of letter.

Note: Acronyms: aOR = adjusted odds ratio; EHR = electronic health records; IRR = incidence rate ratio; RCT = randomized controlled trial; RRR = ratios of risk ratios

Descriptions of study population demographics varied across included studies. Among non-mutually exclusive study population characteristics reported, ten studies noted focusing on increasing influenza vaccination within low-income populations.^43,45,50,58,61,63,67,68,81,88^ Nine studies were implemented among racial and ethnic minority populations, specifically Hispanic, Non-Hispanic Black, and Non-Hispanic American Indian and Alaskan Native adults.^44,47,56,60,62,63,78–80^ Eight studies focused on older aged (≥65 years) populations.^43,51,63,67,68,86,91,93^ Eight studies focused on populations with specific health conditions.^48,69,70,72,74,77,83,84^ Five studies targeted pregnant women.^57,60,79,80,88^ Four studies were among populations who were uninsured or Medicaid recipients.^50,58,61,80^ Three studies were in rural or medically underserved areas.^45,78,85^ Finally, two studies aimed to increase influenza vaccination among persons experiencing homelessness.^45,61^

### Implementation strategies

**Figure 4.**
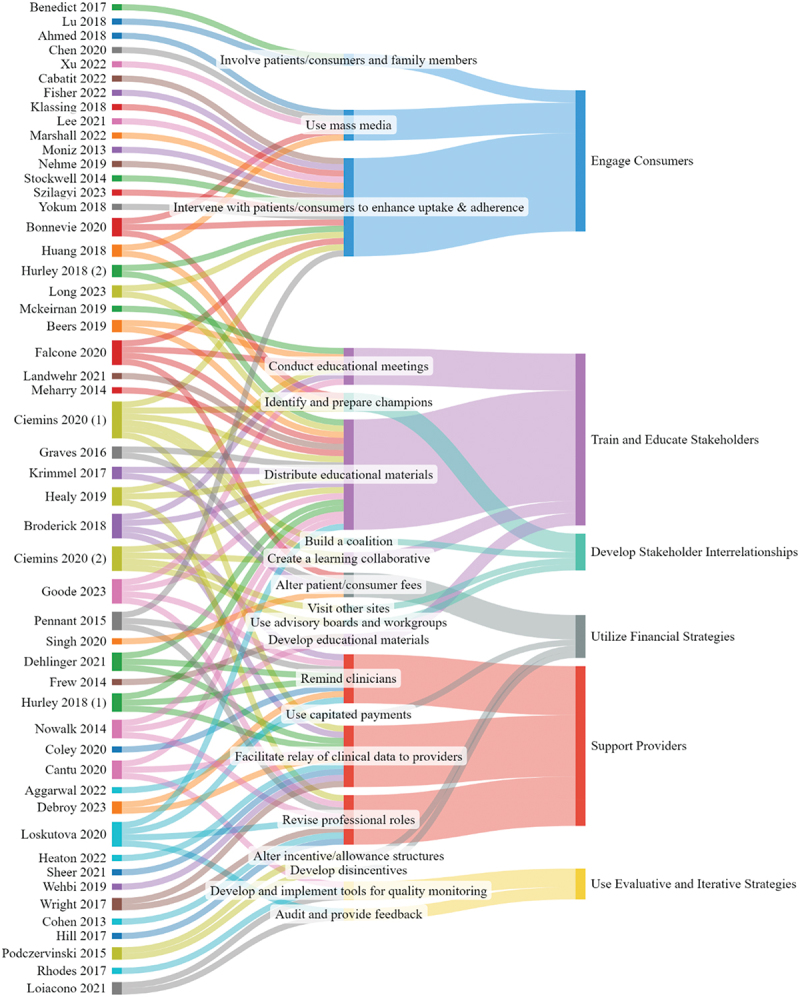
Mapping of studies with implementation strategies and categories from the expert recommendations for implementing change (ERIC).

**Figure 5.**
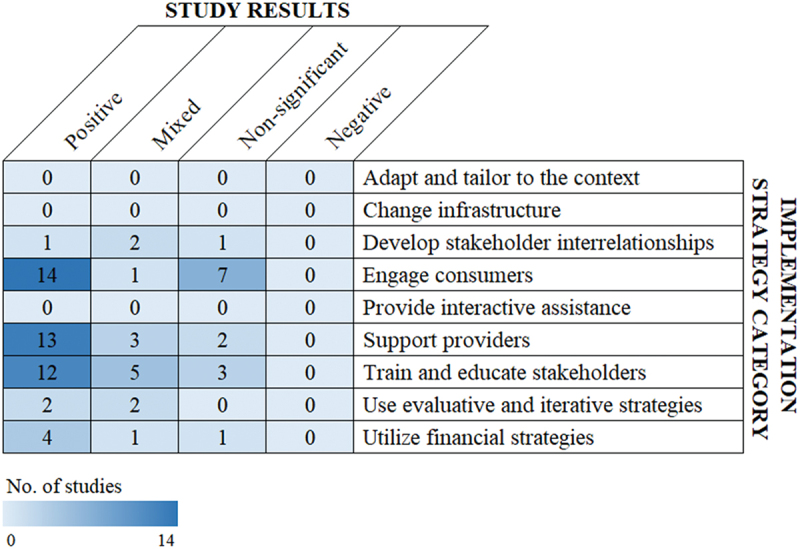
Categories and results of implementation strategies among included studies.

Non-mutually exclusive categories of implementation strategies varied (Figure 4). Among all studies, 22 *engaged consumers*, 20 *trained and educated stakeholders*, 18 *supported providers*, 6 *utilized financial strategies*, 4 *used evaluative and iterative strategies*, and 4 *developed stakeholder interrelationships*. Notably, many studies included in the rapid scoping review were multi-component, bundled interventions encompassing multiple categories of implementation strategies. No studies included in this analysis used implementation strategies designated as *providing interactive assistance, changing infrastructure*, and *adapting and tailoring to the context*. Results from these studies varied across implementation strategy category, with the highest number of studies with positive study findings found for *engaging consumers* (Figure 5). No studies found negative (i.e., decreased vaccination) results.

Many studies encompassed multiple intervention categories.

### Implementation strategies – engage consumers

The most frequently used type of implementation strategy (N = 22) was those that engaged consumers and beneficiaries to increase demand and uptake of seasonal influenza vaccines. Many studies examined the use of patient vaccination reminders and educational messages delivered by text and electronic systems, with varying results. Some studies found significantly higher vaccination among those receiving reminders,^49,58,68,72,77,88^ with one finding that the addition of postal mail reminders along with texts and electronic reminders further increased vaccination.^81^ Notably, two studies exclusively using mailed letters also found an increase in influenza vaccination; however, a third study compared the use of a personal phone call or standardized mailed letter, with non-significant findings.^69,83,93^ Many studies using text or electronic messages also found mixed or non-significant results,^53,59,80,89^ with one finding that while intention to receive a vaccine increased, actual receipt did not.^74^ Another study found that the COVID-19 pandemic contributed substantial barriers to the success of the intervention, including misinterpretation of outreach communications and resource limitations.^59^ In terms of populations with historically lower influenza vaccination rates, two studies noted using electronic reminders for low-income adults, with one demonstrating positive results and another showing mixed findings on increasing vaccination.^67,88^ Another study focused on a primarily racial and ethnic minority, lower educational status, and uninsured population, and had non-significant findings.^80^ Finally, one study focused on motivational mailed letters to older Medicare recipients, with positive results.^93^

Three studies examined the use of social media to engage consumers, with one finding that those who used social media platforms for health information were more likely to be vaccinated,^44^ while a second study had non-significant findings on the use of social media micro-influencers on increasing vaccination rates among Black and Hispanic populations.^47^ The third study enhanced an initial intervention with an additional social media campaign, with positive findings.^66^ Newspaper articles on influenza were found by two studies to increase adult influenza vaccine uptake, notably among older patients.^51,92^

Other studies that engaged consumers focused on direct outreach, with two studies finding that the recommendation and offer of vaccination by a medical provider increased vaccination.^46,76^

### Implementation strategies – train and educate stakeholders

Twenty studies included components that sought to train and educate seasonal influenza vaccine stakeholders, including vaccination implementers (i.e., providers, clinicians, pharmacists) as well as beneficiaries (e.g., patients, employees). Most studies that included provider education showed positive results;^57,58,61,63,75,82^ however, some studies had mixed findings.^45,48^ Two studies that used a learning collaborative approach including distributing educational materials to providers showed mixed results, with some improvement in individual health systems but overall non-significant effects.^52,53^ One study examined pharmacist immunization training among rural, medically underserved American Indian and Alaskan Native populations and found an increase in vaccines administered.^78^ One study looked at a toolkit developed to support a standing order program, and found an overall increase in vaccination, although not among older (≥65 years) adults.^82^

Multiple studies engaging consumers found positive results based on patient posters, visual aids, videos, and information sheets on the benefits of vaccination,^45,50,58,61,63,68,70,75,79^ while others found mixed or non-significant findings.^60,67,74^ In addition to clinical settings, two studies providing educational handouts to employees also showed increases in vaccination.^62,71^ Among studies using visual educational materials, many focused on vulnerable populations including low-income adults, persons experiencing homelessness, Medicaid recipients, uninsured, racial and ethnic minority populations, pregnant women, non-English speakers, and older patients, with no clear patterns identified in intervention success.^45,75,50,75,58,75,61–63,75,68,79^ One study delivered educational messaging on influenza vaccines during the consent form administration process for medical visits, and found higher resulting rates of influenza vaccination.^57^

### Implementation strategies – support providers

Studies that included strategies designed to support providers (N = 18) showed mixed results. Strategies that enabled providers to have greater access to immunization records primarily showed mixed^50,53^ or non-significant results.^56,64^ One study that enabled a bidirectional link between pharmacy-based electronic platforms and state immunization records showed an increase in vaccination rates.^90^ Studies that involved alerts to providers (including on patient eligibility for vaccination) also showed positive findings.^55,57,61,68,75,83,86^ One study involving electronic alerts and weekly e-mails to providers showed an overall reduction in missed opportunities for influenza vaccination; however, no improvements were observed among non-Hispanic Black patients and non-English speakers.^48^ Another study examined pre-visit preventative care planning at nurse practitioner-managed clinics and found higher vaccination compared to national rates.^91^ Three studies that used strategies to revise professional roles or clinical workflow found large increases in vaccination rates compared to prior years or baseline periods.^54,63,65^ Finally, two studies that used standing order programs for vaccination of patients also found significant increases in vaccination rates.^75,82^

### Implementation strategies – utilize financial strategies

Six studies utilized financial strategies to increase seasonal influenza vaccination.^43,58,62,70,84,87^ Four studies offered free vaccinations (two via pharmacy vouchers, one offered within the workplace, one at a free clinic), with all finding significant improvements in vaccination.^58,62,70,87^ Another study offered incentive gifts cards to cancer care center employees and clinical staff for achieving 95% vaccination, finding this strategy to be less effective than a punitive approach (disciplinary action).^84^ Finally, one study looked at the use of capitated payments via Medicare Advantage, finding no significant differences in influenza vaccination compared to the previous year.^43^

### Implementation strategies – evaluative and iterative strategies

Four studies used evaluative and iterative strategies to promote uptake of seasonal influenza vaccines.^50,73,75,85^ One study randomized pharmacies nationwide to receive an electronic report comparing their performance with peers.^73^ The study yielded mixed results, with non-significant findings for additional doses administered in the intervention versus control group but a significant increase in additional doses among historically low-performing pharmacies. Another study implemented tools for quality monitoring of vaccination processes, including a vaccination screening tool and documentation form, resulting in an increase in vaccines administered.^85^ One study used a multicomponent approach that included quarterly performance reports to primary care clinics, finding an increase in influenza vaccination rate by almost 7%.^75^ Finally, another study included quality monitoring tools in its bundled intervention package, finding some sustained improvement in vaccination rate.^50^

### Implementation strategies – develop stakeholder interrelationships

Four studies focused on strategies to develop stakeholder interrelationships.^47,52,53,66^ One study used social media micro-influencers to champion vaccines among a primarily Black and Hispanic audience, with non-significant results in improvements to vaccination rates.^47^ Another study used community health workers at a college campus to champion vaccination, finding a significant increase in vaccination rates among students.^66^ Two studies used learning collaborative approaches to convene expert advisory panels, work across specialties, and conduct site visits, with some improvement in individual health systems but overall non-significant effects.^52,53^

## Discussion

### Strategies, settings, and study populations

In this rapid scoping review, multiple types of implementation strategies to increase uptake and coverage of seasonal influenza vaccines were identified; most frequently, approaches that *engaged consumers* (N = 22), *trained and educated stakeholders* (N = 20), and *supported providers* (N = 18). The highest geographic representation was among states located in the Northeast U.S. region (N = 12). Notably, many states with the lowest (<45%) seasonal influenza vaccine coverage nationwide (e.g., Louisiana, Mississippi, Idaho, Wyoming) were not extensively represented by studies identified in this review.^6^ Details on study population demographics varied widely, with ten noting focusing on low-income populations, nine focused on racial and ethnic minority groups, and eight focused on older aged adults. However, a lack of reporting on geographic and demographic characteristics across all studies limited the ability to connect barriers and facilitators to vaccine uptake for specific populations with effective strategies for improvement. These findings suggest a potential need for improved tailoring of implementation strategies to address local barriers and contextual determinants of influenza vaccination.^28^

### Study results

Among 51 included studies conducted across various study populations, 33 had positive, 11 had non-significant, and 7 had mixed results with respect to increasing influenza vaccination uptake and coverage. However, several study design and methodological issues limit characterization of the evidence on effectiveness of implementation strategies on improving influenza vaccination. While most studies included in the review were experimental, only 35% used randomization procedures, which may reduce the quality of available evidence and limit the ability to perform a systematic review and meta-analysis. Studies also used different outcomes to characterize increase in seasonal influenza vaccination (e.g., odds of vaccination, vaccination rate, number of vaccines administered), making comparisons of magnitude of effect challenging. Additionally, not all studies reported outcomes that included tests for statistical significance, limiting the ability to fully characterize intervention effect. Furthermore, 23 studies used multi-component, packaged interventions, which – in absence of appropriately specified implementation measures – may introduce methodological challenges to characterizing which approaches were most successful at increasing vaccination.^94^

Collectively, these challenges limit the ability to comprehensively highlight promising approaches to increasing influenza vaccination. This analysis suggests additional research with robust methods and high-quality study design that examines both effectiveness and implementation outcomes is needed.^95^ To address gaps in vaccination based on demographic characteristics, researchers should consider prespecified priority study populations and designing implementation strategies in alignment with the specific facilitators and barriers to vaccination for these groups. Approaches such as rapid community assessments could facilitate improved understanding of concerns about vaccination and prioritization of strategies.^96^ For studies encompassing broader populations, subgroup analyses may illuminate differences in results or contextualize overall mixed study findings. Finally, further research is needed to explore the impact of the COVID-19 pandemic and the emergence of other respiratory pathogens on implementation strategies to increase seasonal influenza vaccination, including pan-respiratory public health messaging, coadministration of vaccines, logistical considerations, and changes in public attitudes and perceptions of vaccines.

### Research in context

Prior reviews synthesizing evidence on effectiveness of strategies to increase influenza vaccination underscore heterogeneity in approaches and inconsistent results. Among the limited formal assessments of the evidence, only one review has facilitated development of a Cochrane Clinical Answer for decision-making on appropriate strategies.^97^ This 2018 systematic review of global health studies of interventions to increase influenza vaccination among older (≥60 years) patients found variation among interventions delivered, limiting the ability to perform meta-analyses.^98^ Furthermore, this review noted challenges of evaluating effectiveness of interventions performed at a societal level (e.g., large-scale media campaigns, mass media outreach). However, strategies that increased community demand for vaccines – including patient outreach via reminders, letters, and education, home visits to administer vaccines, provision of free vaccines, payments to physicians, and physician reminders – improved vaccination rates. These results echo findings of this scoping review from one study showing successful engagement of older consumers through mailed motivational letters.^93^

An earlier (2012) review of interventions to improve influenza vaccination among community-dwelling adults of all ages found that patient financial incentives, audit-and-feedback approaches, provider reminders, provider financial incentives, team restructuring, patient outreach, service site change, clinician education to be modestly effective.^99^ In particular, this review highlighted the promise of approaches to allow vaccines to be administered by nurses and other medical personnel, as well as patient outreach involving direct contact (e.g., personalized phone calls). Another review also found substantial evidence that direct outreach and strategies supporting providers could improve vaccination among vulnerable (e.g., low income, racial and ethnic minority, immigrant) groups globally.^100^ The current rapid scoping review found non-significant results among the three studies using phone calls for patient outreach, even when calls were personalized.^59,67,69^ However, all studies included in this review that supported providers by revising professional roles by strategies such as standing order programs,^65,75,82^ provision of vaccines via nurses or pharmacists,^54,65,83,91^ and clinical process and workflow redesign^61,63^ had positive results.

Three systematic reviews of strategies to increase influenza vaccination among pregnant women found that provision of educational materials (e.g., informational pamphlets, face-to-face messaging) could be effective.^101–103^ Studies included in the present review focusing on pregnant women found that education to pregnant women delivered by staff directly,^57^ through provision of pamphlets,^79^ or via text messages^88^ increased vaccination. However, a narrative review of strategies to increase influenza vaccination specifically among Black pregnant women notes mixed results from various approaches, with multi-component interventions including practice-based, group prenatal care, and culturally competent educational messages showing the most success.^104^

Other reviews produced important considerations on effective strategies. One systematic review of hospital-based interventions targeting inpatient influenza vaccine uptake highlighted the promise of standing order programs, particularly when included in multi-component interventions. A rapid review from Australia of strategies to improve influenza vaccination also found that standing order approaches were successful.^105^ These findings are supported by three studies identified in the current scoping review;^65,75,82^ however, one study noted that an increase in vaccination based on standing order programs was not found for older patients.^82^ A scoping review of strategies to increase influenza vaccination among patients with chronic obstructive pulmonary disease also found that multi-component strategies (patient- and provider-focused) were the most effective at increasing vaccination.^106^ Finally, in terms of implementation setting, one review found that pharmacy-based interventions resulted in higher vaccination when compared to routine care.^107^

### Limitations

Several limitations must be considered. First, the rapid scoping review approach reduced dual title and abstract screening of articles, potentially increasing risk of bias in study selection. To address this bias, dual screening was performed on double (40%) the amount of randomly sampled records suggested by Cochrane (20%) for rapid scoping reviews.^35^ An assessment of percent agreement was conducted to confirm high agreement prior to continuing single title and abstract screening of the remaining 60% of records.

Second, a lack of critical appraisal of risk of bias, quality of evidence, and meta-analysis of study results during the scoping review process limited the ability to fully characterize the strength of the evidence supporting an implementation strategy. While results of studies were informally presented, this analysis should not be used to guide recommendations on preferred implementation strategies.

Third, certain eligibility criteria may have reduced the number of studies included that could contribute meaningful insights on promising approaches. Notably, the decision to exclude studies not published in English and to restricted to studies conducted in the United States to reflect uniform vaccination policies recommending that all adults receive seasonal influenza vaccines may have excluded potential useful evidence from global health contexts.^108,109^ Additionally, restricting results to those published in peer-reviewed journals may have eliminated inclusion of implementation strategies performed outside of formal research networks. For example, many implementation strategies to increase influenza vaccination have been implemented by federal, state, or local agencies and healthcare systems.^110–112^ To achieve higher potential for identification of eligible studies, six biomedical databases were searched.

Fourth, substantial heterogeneity in terminology used to define implementation strategies may have omitted otherwise eligible studies. While significant efforts have been made to harmonize terminology within implementation science, much of the research on increasing seasonal influenza vaccination may have been conducted outside of this field.^26,113^ To capture a wide array of implementation approaches, a broad list of search terms was developed in consultation with a medical librarian. Additionally, commonly used ERIC implementation strategies and categories were used to classify results in this scoping review, potentially facilitating future comparisons of results.

## Conclusion

This rapid scoping review provides an overview of the evidence on effectiveness of implementation strategies to increase seasonal influenza vaccination among U.S. adults. Substantial variation in implementation approaches, implementation settings, study populations, study designs, and methods limit the ability to conclude which strategies are most effective at increasing influenza vaccination, particularly for populations with historically lower vaccination. Future studies should consider focusing on key populations to align specific individual and contextual determinants for vaccination with appropriate implementation strategies.

## Supplementary Material

Supplemental Material
