# Supplementary material for: Implementation strategies to increase seasonal influenza vaccination among adults: A rapid scoping review
Source: Hum Vaccin Immunother. 2025 Apr 7;21(1):2481005. doi: 10.1080/21645515.2025.2481005 (PMC11980457; doi:10.1080/21645515.2025.2481005)
Supplement: Supplemental Material [file KHVI_A_2481005_SM1253.docx]

**Supplementary Appendix**

**Supplementary Table 1.** Reporting Items for Systematic reviews and Meta-Analyses extension for Scoping Reviews (PRISMA-ScR) Checklist

| **SECTION** | **ITEM** | **PRISMA-ScR CHECKLIST ITEM** | **REPORTED** |
| --- | --- | --- | --- |
| **TITLE** | | | |
| Title | 1 | Identify the report as a scoping review. | ✓ |
| **ABSTRACT** | | | |
| Structured summary | 2 | Provide a structured summary that includes (as applicable): background, objectives, eligibility criteria, sources of evidence, charting methods, results, and conclusions that relate to the review questions and objectives. | ✓ |
| **INTRODUCTION** | | | |
| Rationale | 3 | Describe the rationale for the review in the context of what is already known. Explain why the review questions/objectives lend themselves to a scoping review approach. | ✓ |
| Objectives | 4 | Provide an explicit statement of the questions and objectives being addressed with reference to their key elements (e.g., population or participants, concepts, and context) or other relevant key elements used to conceptualize the review questions and/or objectives. | ✓ |
| **METHODS** | | | |
| Protocol and registration | 5 | Indicate whether a review protocol exists; state if and where it can be accessed (e.g., a Web address); and if available, provide registration information, including the registration number. | ✓ |
| Eligibility criteria | 6 | Specify characteristics of the sources of evidence used as eligibility criteria (e.g., years considered, language, and publication status), and provide a rationale. | ✓ |
| Information sources* | 7 | Describe all information sources in the search (e.g., databases with dates of coverage and contact with authors to identify additional sources), as well as the date the most recent search was executed. | ✓ |
| Search | 8 | Present the full electronic search strategy for at least 1 database, including any limits used, such that it could be repeated. | ✓ |
| Selection of sources of evidence† | 9 | State the process for selecting sources of evidence (i.e., screening and eligibility) included in the scoping review. | ✓ |
| Data charting process‡ | 10 | Describe the methods of charting data from the included sources of evidence (e.g., calibrated forms or forms that have been tested by the team before their use, and whether data charting was done independently or in duplicate) and any processes for obtaining and confirming data from investigators. | ✓ |
| Data items | 11 | List and define all variables for which data were sought and any assumptions and simplifications made. | ✓ |
| Critical appraisal of individual sources of evidence§ | 12 | If done, provide a rationale for conducting a critical appraisal of included sources of evidence; describe the methods used and how this information was used in any data synthesis (if appropriate). | N/A |
| Synthesis of results | 13 | Describe the methods of handling and summarizing the data that were charted. | ✓ |
| **RESULTS** | | | |
| Selection of sources of evidence | 14 | Give numbers of sources of evidence screened, assessed for eligibility, and included in the review, with reasons for exclusions at each stage, ideally using a flow diagram. | ✓ |
| Characteristics of sources of evidence | 15 | For each source of evidence, present characteristics for which data were charted and provide the citations. | ✓ |
| Critical appraisal within sources of evidence | 16 | If done, present data on critical appraisal of included sources of evidence (see item 12). | N/A |
| Results of individual sources of evidence | 17 | For each included source of evidence, present the relevant data that were charted that relate to the review questions and objectives. | ✓ |
| Synthesis of results | 18 | Summarize and/or present the charting results as they relate to the review questions and objectives. | ✓ |
| **DISCUSSION** | | | |
| Summary of evidence | 19 | Summarize the main results (including an overview of concepts, themes, and types of evidence available), link to the review questions and objectives, and consider the relevance to key groups. | ✓ |
| Limitations | 20 | Discuss the limitations of the scoping review process. | ✓ |
| Conclusions | 21 | Provide a general interpretation of the results with respect to the review questions and objectives, as well as potential implications and/or next steps. | ✓ |
| **FUNDING** | | | |
| Funding | 22 | Describe sources of funding for the included sources of evidence, as well as sources of funding for the scoping review. Describe the role of the funders of the scoping review. | ✓ |

JBI = Joanna Briggs Institute; PRISMA-ScR = Preferred Reporting Items for Systematic reviews and Meta-Analyses extension for Scoping Reviews.

* Where *sources of evidence* (see second footnote) are compiled from, such as bibliographic databases, social media platforms, and Web sites.

† A more inclusive/heterogeneous term used to account for the different types of evidence or data sources (e.g., quantitative and/or qualitative research, expert opinion, and policy documents) that may be eligible in a scoping review as opposed to only studies. This is not to be confused with *information sources* (see first footnote).

‡ The frameworks by Arksey and O’Malley (6) and Levac and colleagues (7) and the JBI guidance (4, 5) refer to the process of data extraction in a scoping review as data charting*.*

§ The process of systematically examining research evidence to assess its validity, results, and relevance before using it to inform a decision. This term is used for items 12 and 19 instead of "risk of bias" (which is more applicable to systematic reviews of interventions) to include and acknowledge the various sources of evidence that may be used in a scoping review (e.g., quantitative and/or qualitative research, expert opinion, and policy document).

*From:* Tricco AC, Lillie E, Zarin W, O'Brien KK, Colquhoun H, Levac D, et al. PRISMA Extension for Scoping Reviews (PRISMAScR): Checklist and Explanation. Ann Intern Med. 2018;169:467–473. [doi: 10.7326/M18-0850](http://annals.org/aim/fullarticle/2700389/prisma-extension-scoping-reviews-prisma-scr-checklist-explanation).

**Supplementary Table 2.** Definition of research question using PICO (Population, Intervention, Comparison, Outcome) framework

| P | Population | Adults ≥18 years in the United States |
| --- | --- | --- |
| I | Intervention | *Clinical intervention*: Administration of any recommended seasonal influenza vaccine product  *Implementation strategy*: Any clinical and/or public health strategies to address suboptimal uptake and coverage of seasonal influenza vaccines |
| C | Comparison | Usual care |
| O | Outcomes | Uptake and coverage of seasonal influenza vaccines |
| *Research question:* | | Among adults in the United States, what is the evidence on the effectiveness of strategies to increase uptake and coverage of seasonal influenza vaccines? |

**Supplementary Table 3.** Rapid scoping review MEDLINE search terms

| **Strategy** |
| --- |
| 1. Influenza Vaccines/ 2. (seasonal ADJ5 (influenza OR flu)).tw. 3. ((influenza OR flu) ADJ5 (immuni* OR vaccin* OR shot OR shots)).tw. 4. 1 OR 2 OR 3 5. exp Adult/ 6. (adult* OR senior* OR elder* OR older).tw 7. 5 OR 6 8. 4 AND 7 9. exp "health disparate, minority and vulnerable populations"/ OR homebound persons/ OR ill-housed persons/ OR exp "social determinants of health"/ OR exp Disabled Persons/ OR exp Demography/ OR exp Sociological Factors/ OR Medically Uninsured/ OR exp "emigrants and immigrants"/ or medically uninsured/ or refugees/ or veterans/ or working poor/ 10. (vulnerable OR vulnerability OR underserved OR uninsured OR dispar* OR equity OR inequit* OR determinant* OR ethnic* OR minorit* OR race OR racial OR socioeconomic* OR SES OR homeless OR ill-housed OR disable* OR disabilit* OR homebound OR housebound OR social factor* OR high-risk OR higher risk OR increased risk OR disadvantage* OR priority population* OR unemploy* OR employment status OR poverty OR income OR low-wage OR single-parent* OR non-English OR speak English OR barrier* OR language spoken OR multi-unit OR multifamily OR mobile home* OR crowding OR crowded housing OR no vehicle OR transportation OR group quarter* OR group housing OR communal living OR neighborhood OR community OR residence OR residential OR census tract*).ti,ab,kf. 11. 9 OR 10 12. 8 AND 11 13. (intervention* OR strateg* OR program* OR plan* OR implement* OR accept* OR access* OR uptake OR increase* OR improv* OR quality OR execute* OR executi* OR application* OR performance OR administration OR organiz* OR disseminat* OR distribut* OR tactic* OR action* OR approach* OR procedure*).ti,ab,hw,kf. 14. 12 AND 13 15. exp United States/ OR ("United States" OR USA OR US OR [Alabama](http://state.1keydata.com/alabama.php) OR [Alaska](http://state.1keydata.com/alaska.php) OR [Arizona](http://state.1keydata.com/arizona.php) OR [Arkansas](http://state.1keydata.com/arkansas.php) OR [California](http://state.1keydata.com/california.php) OR [Colorado](http://state.1keydata.com/colorado.php) OR [Connecticut](http://state.1keydata.com/connecticut.php) OR [Delaware](http://state.1keydata.com/delaware.php) OR [Florida](http://state.1keydata.com/florida.php) OR [Georgia](http://state.1keydata.com/georgia.php) OR [Hawaii](http://state.1keydata.com/hawaii.php) OR [Idaho](http://state.1keydata.com/idaho.php) OR [Illinois](http://state.1keydata.com/illinois.php) OR [Indiana](http://state.1keydata.com/indiana.php) OR [Iowa](http://state.1keydata.com/iowa.php) OR [Kansas](http://state.1keydata.com/kansas.php) OR [Kentucky](http://state.1keydata.com/kentucky.php) OR [Louisiana](http://state.1keydata.com/louisiana.php) OR [Maine](http://state.1keydata.com/maine.php) OR [Maryland](http://state.1keydata.com/maryland.php) OR [Massachusetts](http://state.1keydata.com/massachusetts.php) OR [Michigan](http://state.1keydata.com/michigan.php) OR [Minnesota](http://state.1keydata.com/minnesota.php) OR [Mississippi](http://state.1keydata.com/mississippi.php) OR [Missouri](http://state.1keydata.com/missouri.php) OR [Montana](http://state.1keydata.com/montana.php) OR [Nebraska](http://state.1keydata.com/nebraska.php) OR [Nevada](http://state.1keydata.com/nevada.php) OR "[New Hampshire](http://state.1keydata.com/new-hampshire.php)" OR "[New Jersey](http://state.1keydata.com/new-jersey.php)" OR "[New Mexico](http://state.1keydata.com/new-mexico.php)" OR "[New York](http://state.1keydata.com/new-york.php)" OR "[North Carolina](http://state.1keydata.com/north-carolina.php)" OR "[North Dakota](http://state.1keydata.com/north-dakota.php)" OR [Ohio](http://state.1keydata.com/ohio.php) OR [Oklahoma](http://state.1keydata.com/oklahoma.php) OR [Oregon](http://state.1keydata.com/oregon.php) OR [Pennsylvania](http://state.1keydata.com/pennsylvania.php) OR "[Rhode Island](http://state.1keydata.com/rhode-island.php)" OR "[South Carolina](http://state.1keydata.com/south-carolina.php)" OR "[South Dakota](http://state.1keydata.com/south-dakota.php)" OR [Tennessee](http://state.1keydata.com/tennessee.php) OR [Texas](http://state.1keydata.com/texas.php) OR [Utah](http://state.1keydata.com/utah.php) OR [Vermont](http://state.1keydata.com/vermont.php) OR [Virginia](http://state.1keydata.com/virginia.php) OR [Washington](http://state.1keydata.com/washington.php) OR "[West Virginia](http://state.1keydata.com/west-virginia.php)" OR [Wisconsin](http://state.1keydata.com/wisconsin.php) OR [Wyoming](http://state.1keydata.com/wyoming.php) OR "New England" OR "Mid West*" OR "West Coast" OR "East Coast" OR Appalachia* OR "African American*" OR "Asian American*" OR "Native American*" OR "American Indian*" OR "Mexican American*" OR "Southern Border" ).tw. 16. 14 AND 15 17. Limit 16 to English language 18. Limit 17 to yr="2010-current" |

**Supplementary Table 4.** Data extraction tool

| **Study details** | |
| --- | --- |
| Authors: |  |
| Year published: |  |
| Title: |  |
| Location of study: |  |
| Study dates: |  |
| Study population: |  |
| Study aim(s): |  |
| **Implementation strategies** | |
| Implementation setting: |  |
| Description of strategies: |  |
| **Methods** | |
| Study design: |  |
| Methods: |  |
| Comparator: |  |
| Effectiveness outcome: |  |
| **Results** | |
| Relevant findings (restrict to influenza vaccination): |  |

**Supplementary Table 5.** CFIR-ERIC strategies

| **Category^a^** | **Strategy^b^** |
| --- | --- |
| Use Evaluative and Iterative Strategies | Assess for readiness and identify barriers and facilitators |
|  | Audit and provide feedback |
|  | Conduct cyclical small tests of change |
|  | Conduct local needs assessment |
|  | Develop a formal implementation blueprint |
|  | Stage implementation scale up |
|  | Develop and implement tools for quality monitoring |
|  | Develop and organize quality monitoring systems |
|  | Obtain and use patients/consumers and family feedback |
|  | Purposely reexamine the implementation |
| Provide Interactive Assistance | Centralize technical assistance |
|  | Facilitation |
|  | Provide clinical supervision |
|  | Provide local technical assistance |
| Adapt and Tailor to the Context | Promote adaptability |
|  | Tailor strategies |
|  | Use data experts |
|  | Use data warehousing techniques |
| Develop Stakeholder Interrelationships | Build a coalition |
|  | Capture and share local knowledge |
|  | Conduct local consensus discussions |
|  | Involve executive boards |
|  | Obtain formal commitments |
|  | Promote network weaving |
|  | Use advisory boards and workgroups |
|  | Develop academic partnerships |
|  | Develop an implementation glossary |
|  | Identify early adopters |
|  | Inform local opinion leaders |
|  | Identify and prepare champions |
|  | Model and simulate change |
|  | Organize clinician implementation team meetings |
|  | Recruit, designate and train for leadership |
|  | Use an implementation adviser |
|  | Visit other sites |
| Train and Educate Stakeholders | Conduct educational meetings |
|  | Conduct educational outreach visits |
|  | Develop educational materials |
|  | Shadow other experts |
|  | Conduct ongoing training |
|  | Create a learning collaborative |
|  | Distribute educational materials |
|  | Make training dynamic |
|  | Provide ongoing consultation |
|  | Use train the trainer strategies |
|  | Work with educational institutions |
| Support Providers | Create new clinical teams |
|  | Develop resource sharing agreements |
|  | Facilitate relay of clinical data to providers |
|  | Remind clinicians |
|  | Revise professional roles |
| Engage Consumers | Increase demand |
|  | Intervene with patients/consumers to enhance uptake & adherence |
|  | Involve patients/consumers and family members |
|  | Prepare patients/consumers to be active participants |
|  | Use mass media |
| Utilize Financial Strategies | Access new funding |
|  | Alter incentive/allowance structures |
|  | Alter patient/consumer fees |
|  | Develop disincentives |
|  | Fund and contract for clinical innovation |
|  | Make billing easier |
|  | Place innovation on fee for service lists/formularies |
|  | Use capitated payments |
|  | Use other payment schemes |
| Change Infrastructure | Change accreditation or membership requirements |
|  | Change liability laws |
|  | Change physical structure and equipment |
|  | Change record system |
|  | Change service sites |
|  | Create or change credentialing and/or licensure standards |
|  | Mandate change |
|  | Start a dissemination organization |

^a^ Waltz, T.J., Powell, B.J., Matthieu, M.M. et al. Use of concept mapping to characterize relationships among implementation strategies and assess their feasibility and importance: results from the Expert Recommendations for Implementing Change (ERIC) study. Implementation Sci 10, 109 (2015). https://doi.org/10.1186/s13012-015-0295-0

^b^ Powell, B.J., Waltz, T.J., Chinman, M.J. et al. A refined compilation of implementation strategies: results from the Expert Recommendations for Implementing Change (ERIC) project. Implementation Sci 10, 21 (2015). https://doi.org/10.1186/s13012-015-0209-1

**Supplementary Figure 1.** Screening process

**
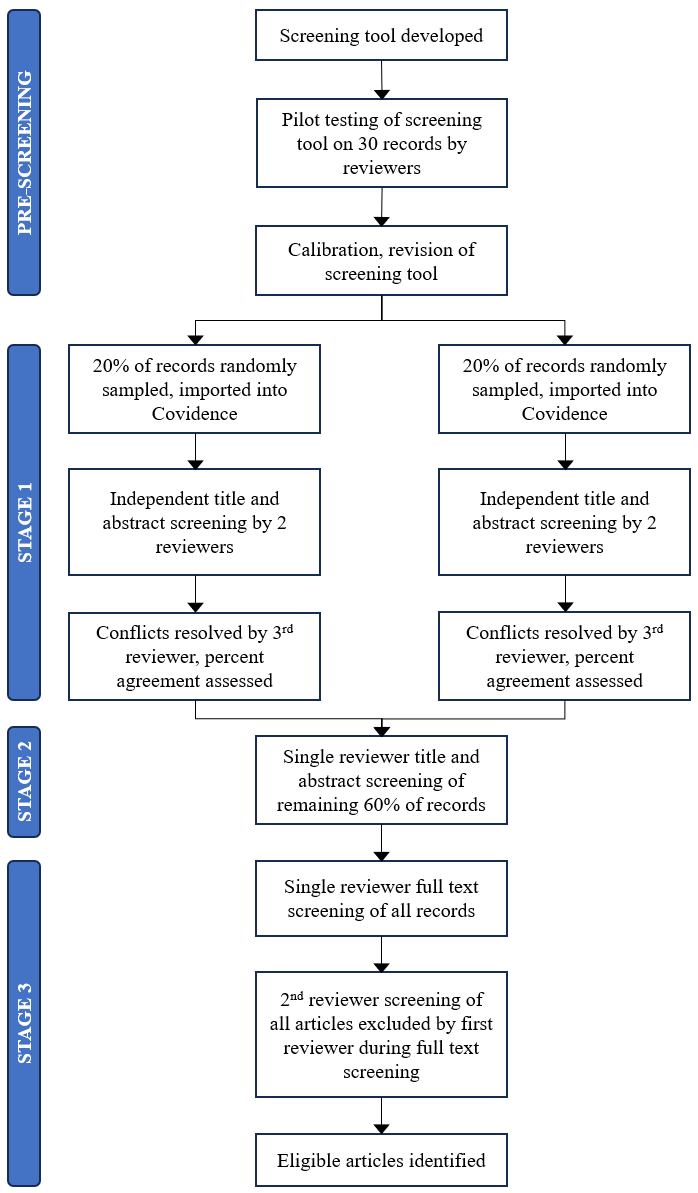
**

**Supplementary Appendix A.** Screening tool

**Project summary**: This scoping review examines the evidence on effectiveness of current implementation strategies to increase uptake of seasonal influenza vaccines in the United States. In 2010, the U.S. Center for Disease Control and Prevention issued a recommendation that all adults receive a seasonal influenza vaccination. In recent years, many approaches have been taken to increase vaccination; however gaps in coverage remain. Characterizing the success of these various strategies is critical to improving their implementation.

**Objective**: To synthesize the evidence on effectiveness of implementation strategies to increase uptake and coverage of seasonal influenza vaccines

**Title and abstract screening guidance:** Please use the below guide to conduct article screening in Covidence based on title and abstracts. These criteria have been developed to rapidly screen relevant studies. Additional criteria will be incorporated at the full text screening phase but will not be used at this stage.

- If the answer to one of the below questions is “yes”, no need to continue with the rest of the questions. Mark the record as “No” (not eligible) in Covidence.
- For title and abstract screening, you do not need to look at the full article for additional information. If you can’t figure out the answer to a question based on the title and abstract, consider the question as “Unclear”, and keep going through the screening questions.

| **Title and Abstract: Screening Criteria** | **YES** | **NO** | **UNCLEAR** |
| --- | --- | --- | --- |
| 1. Is a full **abstract** available for the article? | Continue screening | Continue screening using article title. If any criteria unclear, continue screening. | Continue screening using article title. If any criteria unclear, continue screening. |
| 1. Does the **citation** indicate publication BEFORE February 24, 2010? | Stop screening | Continue screening | Continue screening |
| 1. Does the **title or abstract** indicate the study was conducted or used data that is PRIOR to February 24, 2010?    - Exclude if data overlaps with pre-2010 period (e.g.: 2009-2012). | Stop screening | Continue screening | Continue screening |
| 1. Does the **title or abstract** use a language OTHER than English? | Stop screening | Continue screening | Continue screening |
| 1. Does the **title or abstract** indicate that the study was NOT conducted in the United States?    - Exclude if a review from multiple countries including United States. | Stop screening | Continue screening | Continue screening |
| 1. Does the **title or abstract** indicate that the study was NOT published as a full text article in a peer-reviewed journal?    - Exclude clinical trial registries (example: published in “clinicaltrials.gov” or “trialsearch.who.int”).    - Exclude books.    - Exclude dissertations (example: published in “Dissertation Abstracts International”).    - Exclude conference abstracts.    - Exclude preprints (ex, published in “medRxiv”, “Preprints with The Lancet”).    - Exclude policy briefs. | Stop screening | Continue screening | Continue screening |
| 1. Does the **title or abstract** indicate that the study is NOT focused on seasonal influenza vaccination?    - Exclude if neither title nor abstract specifically mention influenza (or flu).    - Exclude if neither title nor abstract specifically mention vaccination or immunization.    - Exclude if focused on pandemic H1N1 vaccination (2009 A/(H1N1)pdm09, swine flu).    - Exclude if focus is primarily on vaccination against another pathogen (e.g., COVID-19, RSV, pneumococcal, HPV, herpes, hepatitis, meningococcal, shingles). Flu vaccination might be mentioned but it is not the primary focus of the study.    - Include studies looking at preventative care services that include flu vaccines. | Stop screening | Continue screening | Continue screening |
| 1. Does the **title or abstract** indicate that the study was NOT conducted on a human adult population?    - Exclude pediatric, children, infants, adolescents (<18 years). Omit if study targets parents for increasing <18-year-old vaccinations.    - Maternal/motherhood vaccination can be included.    - Exclude animal studies (e.g., mice). | Stop screening | Continue screening | Continue screening |
| 1. Does the **title or abstract** indicate that it was NOT an original research study?    - Exclude study protocols (example: “study protocol” or “protocol for a study” in title).    - Exclude surveillance reports (example: “influenza activity” in title).    - Exclude editorials, commentaries, opinions, letters.    - Exclude guidelines (example: committee recommendations)    - Exclude theoretical frameworks and models.    - Exclude systematic reviews, scoping reviews, literature reviews, meta analyses. | Stop screening | Continue screening | Continue screening |

**Covidence decision: Should this article be included?**

- NO, screening was stopped based on the response of a question.
- MAYBE, one or more screening questions is unclear.
- YES, all screening questions were answered.


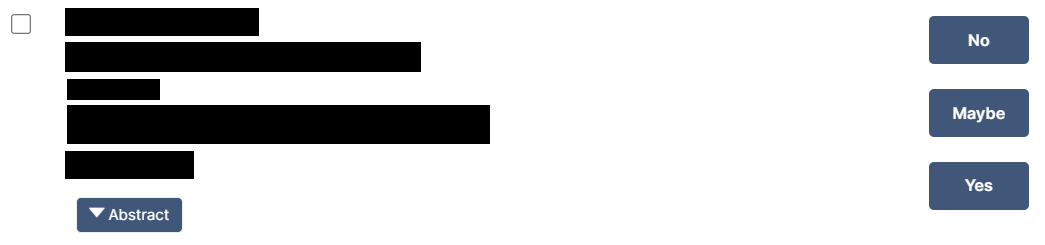


**Additional full-text screening criteria:**

| **Full Text Review: Screening Criteria** | **YES** | **NO** | **UNCLEAR** |
| --- | --- | --- | --- |
| 1. Does the **title, abstract, or full text** indicate that strategies to increase uptake and coverage of seasonal influenza vaccines were NOT studied?    - Include studies with outcomes of odds of vaccination, percentage/proportion vaccinated, vaccination rate, number of vaccines.    - Exclude studies focused exclusively on outcome of willingness to be vaccinated.    - Exclude studies with no comparator (baseline, pre/post, control group, prior year coverage, etc.).    - Exclude molecular, safety, immunogenicity, serology, virology, vaccine efficacy/ effectiveness, and adverse event influenza vaccine studies.    - Exclude studies focused exclusively on cost-effectiveness.    - Exclude qualitative studies with no mixed-methods component.    - Exclude studies focused exclusively on knowledge, attitudes, beliefs, perceptions, perspectives, experiences, determinants, predictors.    - Exclude studies focused on characterizing disparities/gaps/trends in influenza vaccination but no strategy to address vaccination coverage. | Stop screening | Continue screening | Continue screening |
| 1. Does the **title, abstract, or full text** indicate these strategies were NOT actually implemented?    - Exclude strategies that are proposed (e.g., protocols).    - Exclude feasibility studies that do not include an assessment of uptake or coverage. | Stop screening | Continue screening |  |
